# Differential Modulation of Angiogenesis by Erythropoiesis-Stimulating Agents in a Mouse Model of Ischaemic Retinopathy

**DOI:** 10.1371/journal.pone.0011870

**Published:** 2010-07-29

**Authors:** Carmel M. McVicar, Liza M. Colhoun, Jodie L. Abrahams, Claire L. Kitson, Ross Hamilton, Reinhold J. Medina, Dash Durga, Tom A. Gardiner, Pauline M. Rudd, Alan W. Stitt

**Affiliations:** 1 Centre for Vision and Vascular Science, Queen's University Belfast, Belfast, Northern Ireland, United Kingdom; 2 Dublin Oxford Glycobiology Laboratory, The National Institute for Bioprocessing Research and Training, Conway Institute, University College Dublin, Dublin, Ireland; University of Birmingham, United Kingdom

## Abstract

**Background:**

Erythropoiesis stimulating agents (ESAs) are widely used to treat anaemia but concerns exist about their potential to promote pathological angiogenesis in some clinical scenarios. In the current study we have assessed the angiogenic potential of three ESAs; epoetin delta, darbepoetin alfa and epoetin beta using *in vitro* and *in vivo* models.

**Methodology/Principal Findings:**

The epoetins induced angiogenesis in human microvascular endothelial cells at high doses, although darbepoetin alfa was pro-angiogenic at low-doses (1–20 IU/ml). ESA-induced angiogenesis was VEGF-mediated. In a mouse model of ischaemia-induced retinopathy, all ESAs induced generation of reticulocytes but only epoetin beta exacerbated pathological (pre-retinal) neovascularisation in comparison to controls (p<0.05). Only epoetin delta induced a significant revascularisation response which enhanced normality of the vasculature (p<0.05). This was associated with mobilisation of haematopoietic stem cells and their localisation to the retinal vasculature. Darbepoetin alfa also increased the number of active microglia in the ischaemic retina relative to other ESAs (p<0.05). Darbepoetin alfa induced retinal TNFα and VEGF mRNA expression which were up to 4 fold higher than with epoetin delta (p<0.001).

**Conclusions:**

This study has implications for treatment of patients as there are clear differences in the angiogenic potential of the different ESAs.

## Introduction

Hypoxia-mediated secretion of erythropoietin (EPO) from the kidney maintains erythrocyte mass through inhibition of apoptosis in bone marrow precursors. EPO normally occurs at sustained levels within the low picomolar range (1–7 pmol/L) and, while levels can increase by more than 100-fold in severe hypoxia, a negative feedback loop maintains plasma concentrations. As an example of molecular versatility, EPO can also function in a paracrine/autocrine manner to promote tissue survival during ischaemic, toxic and traumatic insults [1]. EPO is expressed in many organs where it can activate the EPO receptor (EPO-R), sometimes in combination with the β-common receptor (βCR; CD131) [2]. This tissue protective role is exemplified during ischaemic brain injury in which hypoxia inducible factor (HIF-1α) regulates EPO expression and serves to reduce infarct size [3]. Importantly, it has been demonstrated that administration of exogenous human recombinant rHuEPO to pre-clinical models of ischaemic injury can significantly reduce neurodegeneration by preventing neuro-glial apoptosis [4].

The anti-apoptotic neuroprotective properties of EPO in ischaemic tissues may occur concomitantly with angiogenesis including promotion of revascularisation [5], [6] and microvascular remodelling [7]. The mechanism for EPO-mediated angiogenesis is complex but includes phosphorylation of eNOS [8], [9] and mobilisation of endothelial progenitor cells (EPCs) with their recruitment to sites requiring vascular repair [10]. EPO-mediated angiogenesis significantly improves wound repair [11], microvascular re-modelling following myocardial infarction [12] and focal cerebral ischaemia [13].

Exogenous EPO may also have important benefits in retinal disease and its neuroprotective activity is shown by inhibition of apoptosis following a variety of cellular insults [4], [14], [15], [16], [17]. In an acute model of hyperoxia-induced retinal ischaemia, administration of exogenous EPO prior to the hypoxic insult can protect neurons, prevent vessel dropout and subsequently suppress the stimulus for hypoxia-induced neovascularisation [18]. However, the phase of disease during which EPO is introduced may alter the outcome and, in the same model, EPO treatment whilst the retina is experiencing hypoxia may enhance pathological, pre-retinal neovascularisation [18]. Conversely, in the same murine model, inhibition of Epo mRNA using interference RNA [19] or blocking the action of EPO-R with soluble receptor [20] proved effective in suppressing retinal neovascularisation. Although EPO therapy may have neuroprotective benefits in certain eye diseases, such treatment needs to be used judiciously.

Erythropoiesis stimulating agents (ESAs) are widely used clinically. Their half lives and therefore their efficacy, are dependent on the heavily sialylated N-linked glycans which protect the protein from proteases and prevent rapid removal from the serum by the asialoglycoprotein receptor. Epoetin delta and epoetin beta each contain four N-linked glycans (Asn24IleThr; Asn38IleThr; Asn83SerSer) while epoetin alpha has been engineered to contain two additional glycosylation sites (Asn57GlyThr; Asn115LysThr). The particular structures of the oligosaccharides are site specific and also depend on the particular glycosylation processing pathways in each cell type. Epoetin delta contains only human oligosaccharides which differ in some ways from other ESAs expressed in CHO cells[21]. For example, CHO-cell-derived epoetins display low levels of N-glycoylneuraminic acid which is not present on sugars attached to Epoetin delta because the human cell line lacks the necessary enzyme and monosaccharide to carry out this step. Epoetin delta has demonstrated efficacy for treatment of anaemia in patients with chronic kidney disease whilst not inducing generation of anti-EPO antibodies [22]. Beyond its erythrogenesis properties, epoetin delta potential to induce tissue responses has not been closely evaluated even though it has potential to interact quite differently with EPO-receptors than other ESAs.

ESAs are mostly used to treat patients with anaemia but in recent years there has been concern that recombinant EPOs have potential to promote pathological angiogenesis in some clinical settings. For patients with diabetic nephropathy or for those who develop anaemia following chemotherapy, activation of EPO-Rs with ESAs could promote accelerated retinopathy or tumour vascularisation respectively. Therefore it is important to evaluate the different ESAs used to treat anaemia and determine their potential to promote angiogenic pathology [23], [24]. In the current study we have evaluated three commonly used ESAs and assessed their angiogenic potential *in vitro* and in an established model of ischaemia-induced proliferative retinopathy. We describe how the angiogenic potential of the human cell-derived Epoetin Delta differs from recombinant EPOs darbepoetin alfa, epoetin beta derived from CHO cells and that these ESAs also have differential impacts on ischaemic retina.

## Methods

### Normal-phase HPLC and N-glycans

Samples were reduced and alkylated before being set into SDS-PAGE gel blocks, washed and digested with PNGase F, (Prozyme, San Leandro, CA, USA) for 16 hours as previously described by Royle *et al* *[25]* The eluted glycans were labelled with 2-AB using the LudgerTag™ 2-AB kit according to the manufactures instructions. Normal-phase HPLC (NPHPLC) was performed using a TSKgel Amide-80 5 µm (250×4.6 mm) column, Waters Alliance 2695 Separations Module with a Waters 2475 Multi Wavelength Fluorescence Detector (λex  = 330 nm and λem  = 420 nm) (Waters Corporation, Millford, MA, USA). A 180 min gradient of 50 mM ammonium formate buffer, pH 4.4 and acetonitrile was used for glycan separation as previously described by Royle *et al* *[25]*. The systems were calibrated by running an external standard of 2AB–dextran ladder (2AB–glucose homopolymer, Ludger) alongside the sample runs.

### ESAs and in vitro angiogenesis

The *in vitro* angiogenesis assay employed was a three dimensional model of angiogenesis as previously described [26]. Human dermal microvascular cells (HDMEC) (Promocell, UK) were suspended in an extracellular matrix gel (Matrigel, Becton Dickinson, England). The HDMECs were cultured in endothelial cell growth medium MV (Promocell, UK) and supplement growth factors as recommended by Promocell.

HDMECs were plated in 30 µl circular spots of 50% Matrigel diluted in medium until they formed endothelial tubular networks around 48 hours post-seeding. Three different ESAs were then added to a second layer of Matrigel superimposed on the primary culture spots. The ESAs studied were epoetin delta (Dynepo), darbepoetin alfa (Aranesp) and epoetin beta (Neorecormon) (0.1–100 IU/ml). The calculation for equivalent doses of ESAs was based on the fact that 200 IU of epoetin delta contains the same peptide mass as 1 µg of darbepoetin alfa [27].

After a further 24 hours the number of endothelial sprouts that had crossed the interface between the two layers were counted around the entire circumference of each primary culture spot using phase contrast microscopy. Angiogenic sprouting from the primary to secondary gel layers was assessed for 9 spots/treatment group. To confirm that autocrine VEGF secretion underlies the angiogenic actions of EPO, the above experiment was repeated with additional treatment groups for each ESA in which the ESA was mixed with a VEGF neutralising antibody at 0.4 mg/ml (Ranibizumab/Lucentis, Novartis, UK) VEGF (40 ng/ml) was employed as a positive angiogenic control.

### Oxygen-Induced Retinopathy Model

All experiments conformed to UK Home Office regulations (project licence No. 2611) and were approved by Queen's University Belfast Ethical Review Committee for Animal Research. Oxygen-induced retinopathy (OIR) was conducted in C57/BL6 wild type mice according to the protocol of Smith *et al.* [28]. In this model, 7-day-old (P7) mouse pups and their nursing dams were exposed to 75% oxygen (humidified medical grade oxygen controlled by a PROOX oxygen controller model 110; Reming Bioinstruments Co. Redfield, NY) for 5 days causing vaso-obliteration and cessation of development of the central retinal capillary beds. On postnatal day 12 (P12) the mice were returned to room air, after which there was acute retinal ischaemia in the avascular regions of the central retina, followed by a potent pre-retinal neovascular response between P15 and P21.

A total of 74 mice were divided into ten groups. Group 1 consisted of P12 controls (n = 4) and was used to confirm that consistent central vasobliteration occurred following hyperoxia exposure. Groups 2, 3, & 4 received daily intraperitoneal (i.p.) injections of low dose treatment of ESAs from P12-P16 inclusive (n = 7 pups/group). The low dose regime for group 2 was epoetin delta (30 iu/Kg), while for group 3 it was equivalent dose of darbepoetin alfa (0.15 µg/Kg) and for group 4 it was epoetin beta (30 iu/Kg). For high-dose ESA treatments, group 5 received epoetin delta (2500 iu/Kg) (n = 14); group 6 received darbepoetin alfa (12.5 µg/Kg) (n = 7) and group 7 received epoetin beta (2500 iu/Kg) (n = 7).

For controls, group 8 (n = 7) received i.p. formulation buffer (sham control for epoetin delta), Group 9 (n = 7) received PBS (sham control for darbepoetin alfa and epoetin beta). The animals from groups 2–9 were sacrificed at P17 with sodium pentobarbital. In addition another group of mice (group 10) received the high dose of epoetin delta 2500 IU/Kg after which they were sacrificed at P23 (n = 7).

### Blood reticulocyte number

Haematocrit levels were quantified using the Sysmex SC9500 analyser (Japan). In addition, reticulocyte counts were conducted since these cells are present in blood for ∼48 hours before developing into mature red blood cells. Reticulocytes represent an appropriate evaluation for the acute experimental system employed. Blood was obtained from each pup by cardiac puncture and placed in EDTA coated tubes. This was then mixed with brilliant cresyl blue and incubated for 20 mins at 37°C. The blood and stain was then smeared onto a glass slide and the number of reticulocytes assessed using a ×100 oil objective lens.

### Microvascular pathology

One eye from each pup (n = 7 per treatment) was enucleated and immediately fixed in 4% PFA. Retinal flat mounts were stained with isolectin B4 (Sigma), co-stained with glial fibrillary acidic protein (GFAP) (Dako) and with the corresponding secondaries streptavidin Alexa Fluor 488 (Molecular Probes) and Alexa Fluor 568 goat anti-rabbit IgG. DAPI (Sigma) was also added to locate the nuclear layers of the retina. Stained retinas were visualised and imaged using confocal microscopy (Eclipse TE2000-U, Nikon, Japan). Avascular and pre-retinal neovascularisation were quantified using Lucia Version 4.60 software as previously described [29].

For some eyes, serial sections of the flat-mounts were cryosectioned at 12 µm thickness (Leica, CM 1900 UV). Every other section was stained with haematoxylin & eosin (H&E) providing a total of 176×12 µm serial sections. All odd number sections were stained with H&E and all the even numbers were left as fluorescent images. All fluorescent sections were imaged using a confocal microscope. These serial sections were cut from the peripheral retinal edge towards the optic nerve in the centre of the retina. As each section transacted the full width of the quadrant and was 12 µm in thickness the exact location of the section could be mapped to the low magnification (x4) images of the flatmount preparation. By examining each section in sequence and correlating with the distance cut into the quadrant it was possible to conclude exactly which region of the flat mount that was being assessed in cross section.

Sca-1 (BD Pharmingen), a marker for haematopoietic stem cells was co-labelled with isolectin B4 with the secondary antibody Alexa Fluoro 568 donkey anti-goat (Invitrogen) in both P17 and P23 retinas (n = 7).

IBA1 (marker for microglia, Abcam) was co-stained with isolectin B4 with the secondary antibody Alexa Fluoro 568 donkey anti-goat (Invitrogen). Microglia were divided into four basic morphological categories as described by Kettenmann et al. [30]. The number and the activation of the microglia were assessed in the ischaemic area of the retina. Four Z-series images were collected per specimen at ×40 using the Nikon Eclipse TE2000-U Confocal Microscope C. Five specimens per treatment were assessed.

### Ultrastructural evaluation

Transmission electron microscopy (TEM) was used to evaluate ultrastructural changes to the retina with high dose of epoetin delta treatment. Flat-mounted retinae were post-fixed in fresh 2.5% glutaraldehyde in 0.1 M sodium cacodylate buffer at 4°C for a minimum of 12 hrs and then further fixed in 1% osmium tetroxide. Tissues were then processed through graded ethanol and embedded in Spurr's resin. Semi-thin sections were prepared, stained with toluidine blue and retinal orientation evaluated in accordance with the flat-mounts previously viewed using confocal microscopy. Ultrathin sections were cut and floated onto 200 mesh copper grids (Agar Scientific Ltd. UK), counter stained with uranyl acetate and lead citrate. Sections were viewed on a Hitachi H-7000 transmission electron microscope.

### Bone marrow analysis

The femurs from each pup were removed and the marrow was flushed from the femurs and stained either with: FITC-conjugated anti-mouse Ly-6A/E (Sca-1) or the appropriate isotype IgG control (all obtained from eBiosciences) for 45 mins at 4°C. The cells were then washed in PBS and spun at 385 g. 10,000 cells were assessed for each marker and the percentage of positive cells was calculated using a Becton Dickinson FACSCalibur.

### Quantitative RT-PCR (qPCR)

Freshly dissected mouse retinas from each HD-EPO-treated experimental group (n = 7 mice/group) were initially placed in RNA Later (Sigma) and stored at −20°C. Total RNA was subsequently extracted and purified (RNeasy Mini Kit; Qiagen, Crawley, UK) with residual DNA removed by DNase I digestion (Qiagen). The quantity and quality of RNA in each sample was determined spectrophotometrically (Nanodrop Technologies, UK). Equal amounts of RNA were reverse transcribed into cDNA (Omniscript Reverse Transcription, Qiagen UK). Real-time quantitative RT-PCR (qPCR) was performed using murine sequence-specific primers for VEGF, TNF-alpha and IL-10. ARP was used as a housekeeping expression control as previously described [31]. Primer sequences are shown in table 1.

**Table 1:** Primer sequences for murine sequence-specific primers for housekeeping gene ARP, VEGF, TNF-alpha and IL-10.

	Forward	Reverse
ARP	5′CGACCTGGAAGTCCAACTAC3′	5′ATCTGCTGCATCTGCTTG3′
VEGF	5′TTACTGCTGTACCTCCACC3′	5′ACAGGACGGCTTGAAGATG3′
TNF-α	5′CCTCACACTCAGATCATCTTC3′	5′CGCTGGCTCAGCCAC TCC3′
IL-10	5′AGCAGCCTTGCAGAAAAGAG3′	5′TAGGAGCATGTGGCTCTGG3′

The PCR reaction mix contained 2 µl of cDNA, 0.3 µmol/L of forward and reverse primer each, 10 µl of Quantitect SYBR Green I mix (Qiagen) and 3.4 µl of RNAse free water making a total reaction of 20 µl. The PCR conditions were as follows: Denaturation, 95°C for 15 min; Amplification (45 cycles), 94°C for 15 s, 61°C for 30 s, and 72°C for 15 s; For normalization of expression data, a 109-bp fragment of the housekeeping ARP mRNA was employed as previously described [31].

## Results

### Glycan profiling

**Figure 1:**
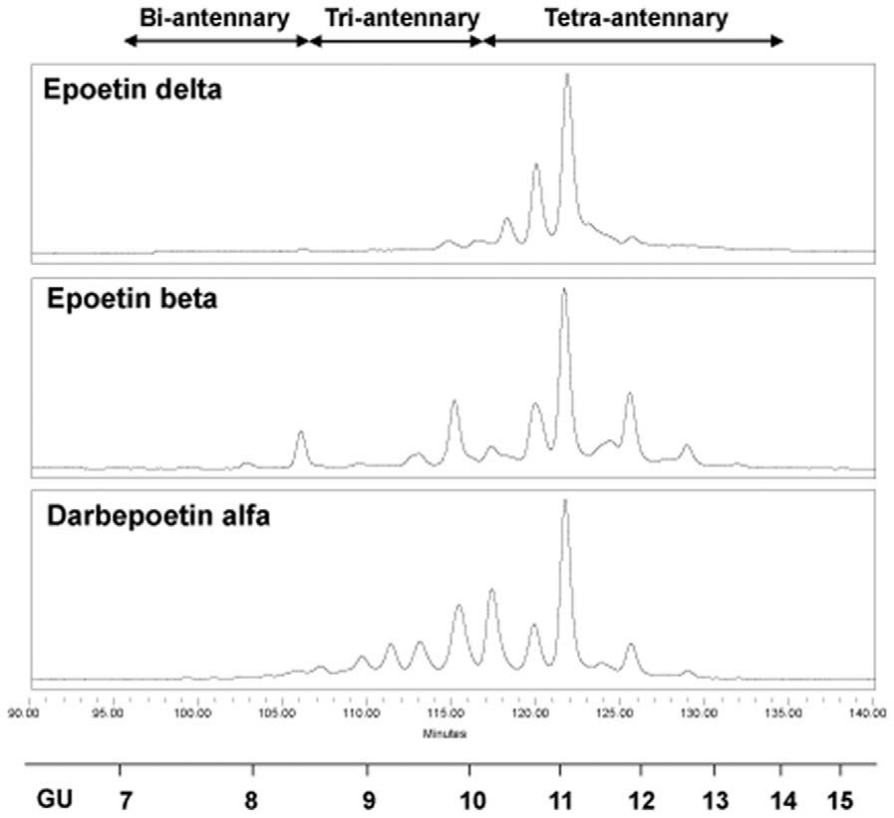
Glycan profiling. Normal phase HILIC profiles of N-linked glycans released from epoetins and labelled with 2-aminobemzamide. The sections of the profiles where bi-, tri- and tetra-antennary glycans elute are indicated at the top of the figure. The elution positions are shown against a scale of glucose oligomers used to calibrate the column.

The N-glycan pools were released from the three epoetins using PNGase F and, after fluorescent labelling with 2-aminobenzamide, were run on a HILIC HPLC column prior to detailed sequencing (Figure 1). The profiles obtained on these high resolution columns are a visual illustration of glycosylation differences between the three ESAs This indicates significant changes in the levels of bi- tri- and tetra-antennary glycans, in sialylation and in fucosylation. The profile obtained for Epoetin delta has a predominant proportion of sialylated core-fucosylated tetra-antennary glycans, reflected in the higher GU values. A full N-glycan profiling for the different Epoetins is currently under study (Shahrokh et al., unpublished results).

### ESA-mediated angiogenesis *in vitro*

Initially the effect of epoetin delta (0.1–100 IU/ml) was determined using the *in vitro* angiogenesis model in HDMECs as described by Stitt *et al.* 2005[26]. Briefly, the in vitro assay was a novel three-dimensional model of angiogenesis that used HDMECs in an extracellular matrix gel. Next day, the second layer of matigel was added with the ESA derivative. After a further 24 hours the number of endothelial sprouts that had crossed the interface between the two layers were counted around the entire circumference of each primary culture spot using phase contrast microscopy.

**Figure 2:**
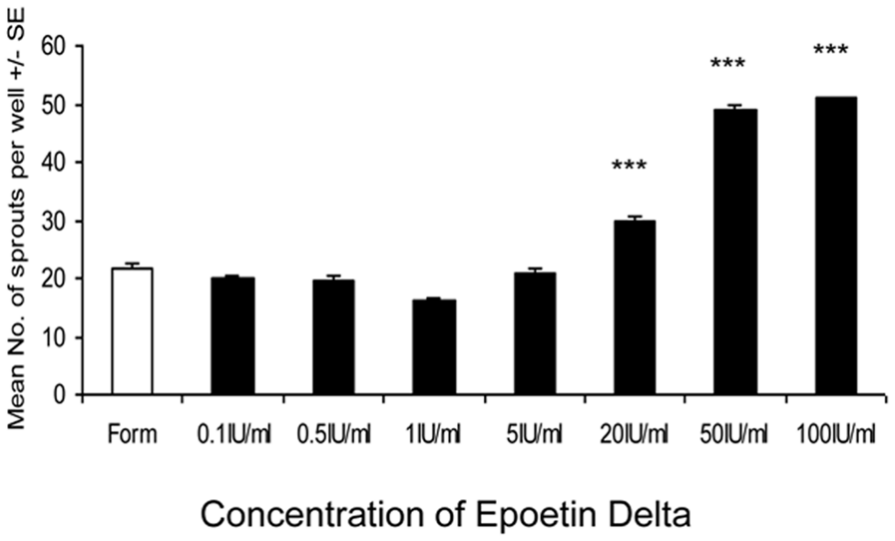
The effect of Epoetin delta on angiogenesis in vitro. The effect of epoetin delta was determined using the *in vitro* angiogenesis model in primary HDMEC cells. At doses >20 IU/ml there is a significant increase in angiogenesis (n = 6). Doses <20 IU/ml is not angiogenic. (Error bars  =  Standard Error of the Mean ***p<0.001.

**Figure 3:**
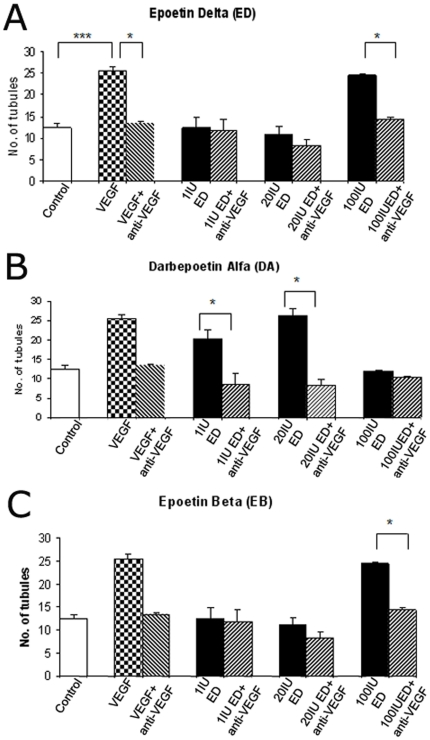
VEGF dose response and prevention of EPO-induced angiogenesis by blockade of VEGF bioavailability. (A) In primary HDMEC cells VEGF was used as a positive control and the anti-VEGF drug Lucentis reduced the angiogenic effect of Epoetin Delta (ED) at 100 IU/ml in tubules grown from primary HDMEC cells. It had little effect on 1 and 20 IU/ml. (B) Darbepoetin Alfa (DA) was considerably more angiogenic than at the 1 and 20 IU/ml doses (p<0.001) although 100 IU failed to induce angiogenesis. (C) Only Epoetin Beta (EB) at 100 IU/ml dose was pro-angiogenic. (n = 6) (Error bars  =  Standard Error of the Mean) * = p<0.05.

This ESA induced no significant angiogenic response with doses at <20 IU/ml however at higher doses (>20 IU/ml) there was a significant, stepwise increase in angiogenesis (p<0.001) (Figure 2). This established that a dosing regime of 1, 20 and 100 IU/ml was appropriate for subsequent experiments. VEGF (40 ng/ml) as an appropriate positive control induced significant angiogenesis in HDMEC cells (p<0.001) (Figure 3). The VEGF neutralising antibody (Ranibizumab at 0.4 mg/ml) prevented VEGF bioactivity and significantly reduced VEGF-mediated angiogenesis to negative control levels (Figure 3).

Epoetin delta induced angiogenesis in the HDMEC cells when compared to controls (p<0.05) (Figure 3A). Darbepoetin alfa at an equivalent dose was considerably more pro-angiogenic at the 1 and 20 IU/ml doses (p<0.001) although 100 IU/ml failed to induce angiogenesis (Figure 3B). A biphasic angiogenic effect was observed with Darbepoetin Alfa in HDMEC primary cells and Human Microvascular Endothelial Cells (HMEC-1 cell line, data not shown). Epoetin beta was similar to epoetin delta in that only the 100 IU/ml dose was pro-angiogenic (Figure 3C). Ranibizumab significantly attenuated the angiogenic effect of all three ESAs (Figure 3A–C).

### Erythrogenesis and acute ESA-treatment in neonatal mice

**Figure 4:**
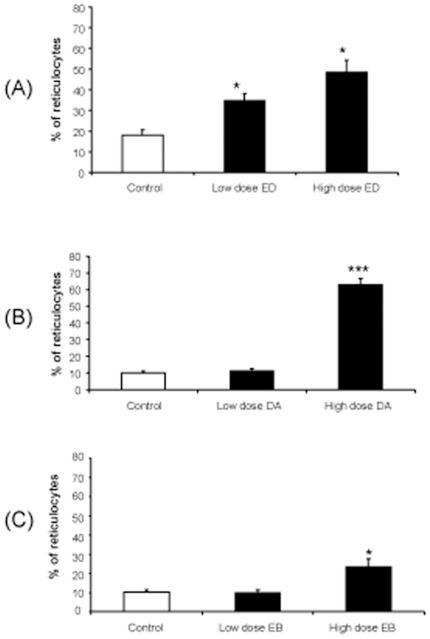
Percentage of reticulocytes as an early indicator for erythrogenesis. The percentage of reticulocytes were analysed as an early indicator for erythrogenesis and these cells were significantly increased by low dose of Epoetin Delta and high dose of Epoetin Delta (A) when compared to control animals (n = 6)) (*p<0.05). Darbepoetin Alfa (B) (***p<0.001) and Epoetin Beta (C) (*p<0.05) only altered the percentage of reticulocytes with the high dose of EPO. (Error bars  =  Standard Error of the Mean).

The haematocrit in epoetin delta, darbepoetin alfa or epoetin beta-treated mice showed no alteration when compared between dosing levels and vehicle controls (data not shown). However, the percentage of reticulocytes was also analysed as an early indicator for erythrogenesis and these cells were significantly increased by both low dose and high dose epoetin delta when compared to control animals (p<0.05). Darbepoetin alfa and epoetin beta only altered the percentage of reticulocytes with the high dose (HD)-EPO regime (Figure 4).

### ESA regulation of pre-retinal neovascularisation

**Figure 5:**
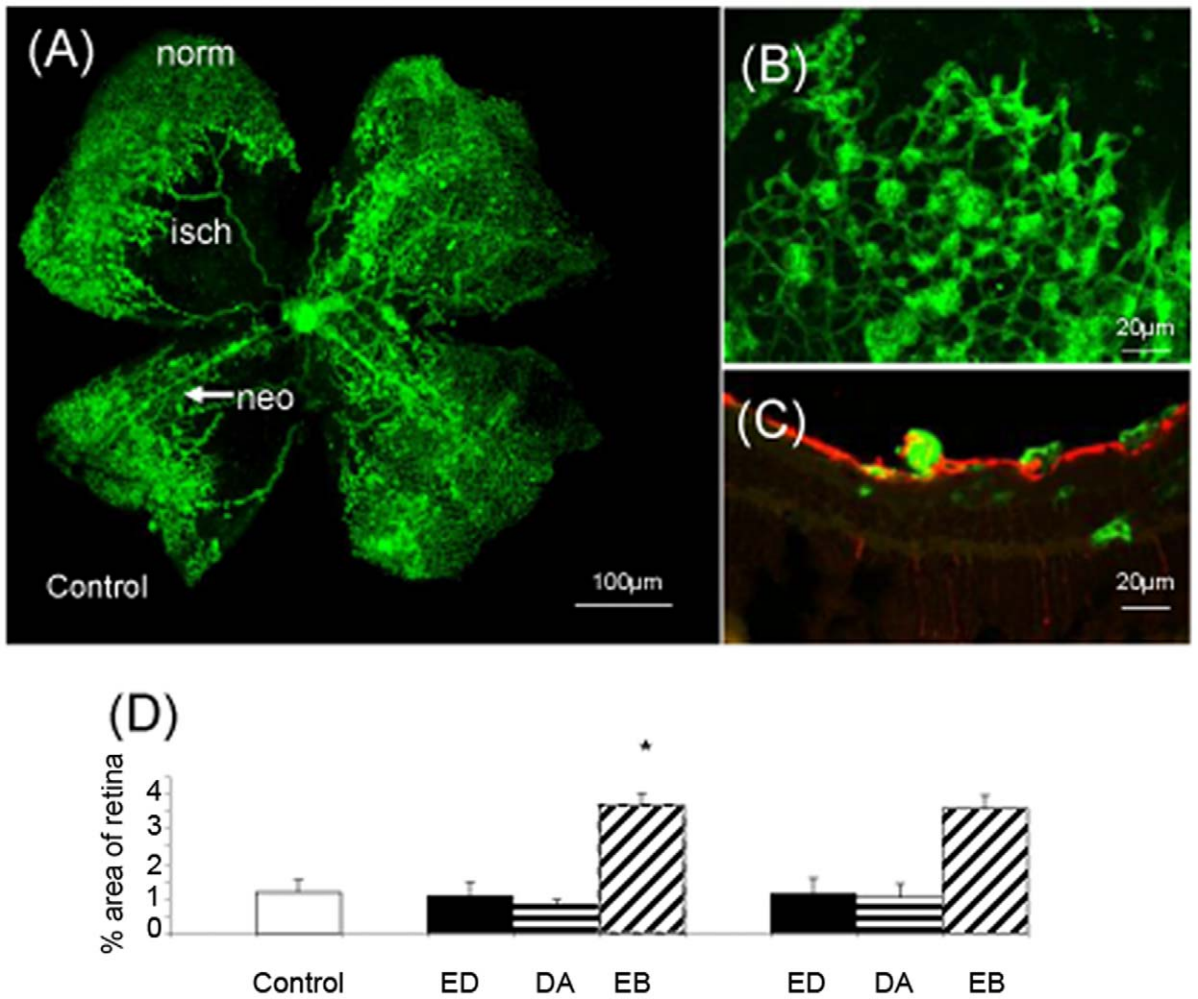
Epoetin delta and Darbepoetin Alfa does not alter pre-retinal neovascularisation in the OIR model. Representative flat-mount of the retina following OIR in a control sample. The typical ischaemic (isch) area of the retina is traced while the normal vasculature (norm) and neovascularisation (neo) is also represented (A). A typical cluster of vascular structures represented as “neovasculature” on a flat-mounted retina (B). When this flat mount is sectioned it shows that these vessels are on top of the retina (C). Epoetin Delta (ED), Darbepoetin Alfa (DA) or Epoetin Beta (EB) treatment of pups with OIR from P12–P17 at low or high dose illustrated that Epoetin Delta and Darbepoetin Alfa did not influence pre-retinal neovascularisation, irrespective of dosing regime, however there was an increase in pre-retinal neovascularisation at both low and high doses of Epoetin Beta (D) (Error bars  =  Standard Error of the Mean). This data has implications for usage of these Erythropoietic stimulating agents (ESAs) in anaemic patients who could have enhanced risk of tumour neovascularization, rheumatoid arthritis and proliferative retinopathy. From this study our data shows that only Epoetin Beta increased the pathological neovascularization. ESAs induce angiogenesis in ischaemic retinopathy but vary in terms of promoting normal retinal perfusion and activation of ischaemic-linked pro-inflammatory responses by the retina.

Consistent with previous reports, OIR induced a temporal pattern of central retina vascular insufficiency upon return to room air at P12 which lead to a reproducible pre-retinal neovascularisation observable on flat-mounts Figure 5 (B) and verified in transverse sections as being on top of the internal limiting membrane (ILM) (Figure 5 (C). Pre-retinal neovascularisation was quantified on flat-mounted retina at P17 (Figure 5B). Epoetin delta or darbepoetin alfa treatment of pups with OIR (treated daily P12–P16) did not influence pre-retinal neovascularisation, irrespective of low or high-dose regime. By contrast epoetin beta (treated daily P12–P16) caused significant enhancement of pre-retinal neovascularisation when compared to controls (Figure 5D).

### ESA treatment and retinal ischaemia and remodelling

**Figure 6:**
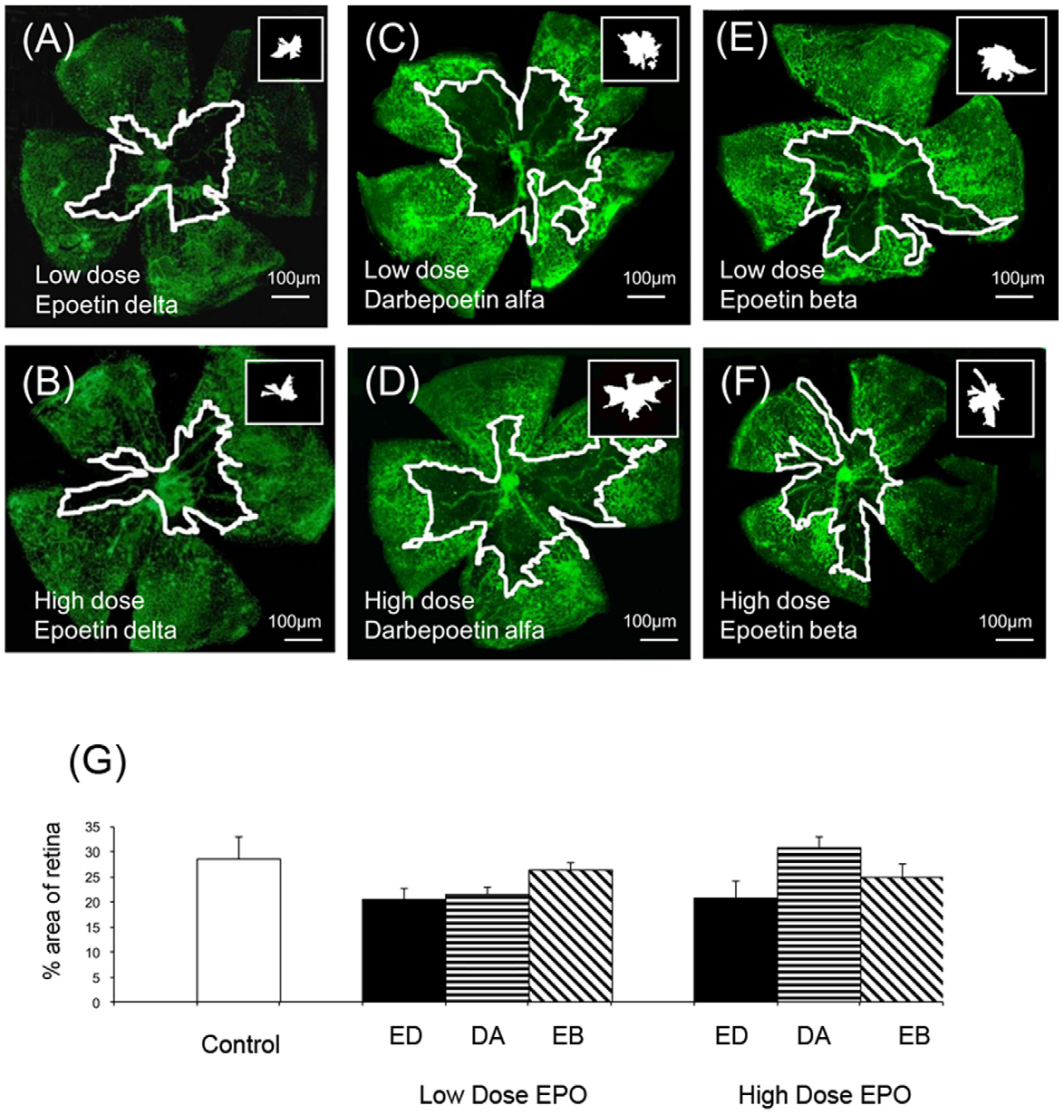
Representative flat-mounts of the retinas following OIR treated with the three ESAs. Flat-mounted retina shows the ischaemic areas highlighted following treatment with low dose of epoetin delta (ED) (A) darbepoetin alfa (DA) (B) epoetin beta (EB) (C) and high dose of epoetin delta (D) darbepoetin alfa (E) and epoetin beta (F). Epoetin Delta, Darbepoetin Alfa or Epoetin Beta treatment of pups with OIR from P12–P17 at low or high dose had no significant influence on the extent of retinal ischaemia (avascular area) (G).

**Figure 7:**
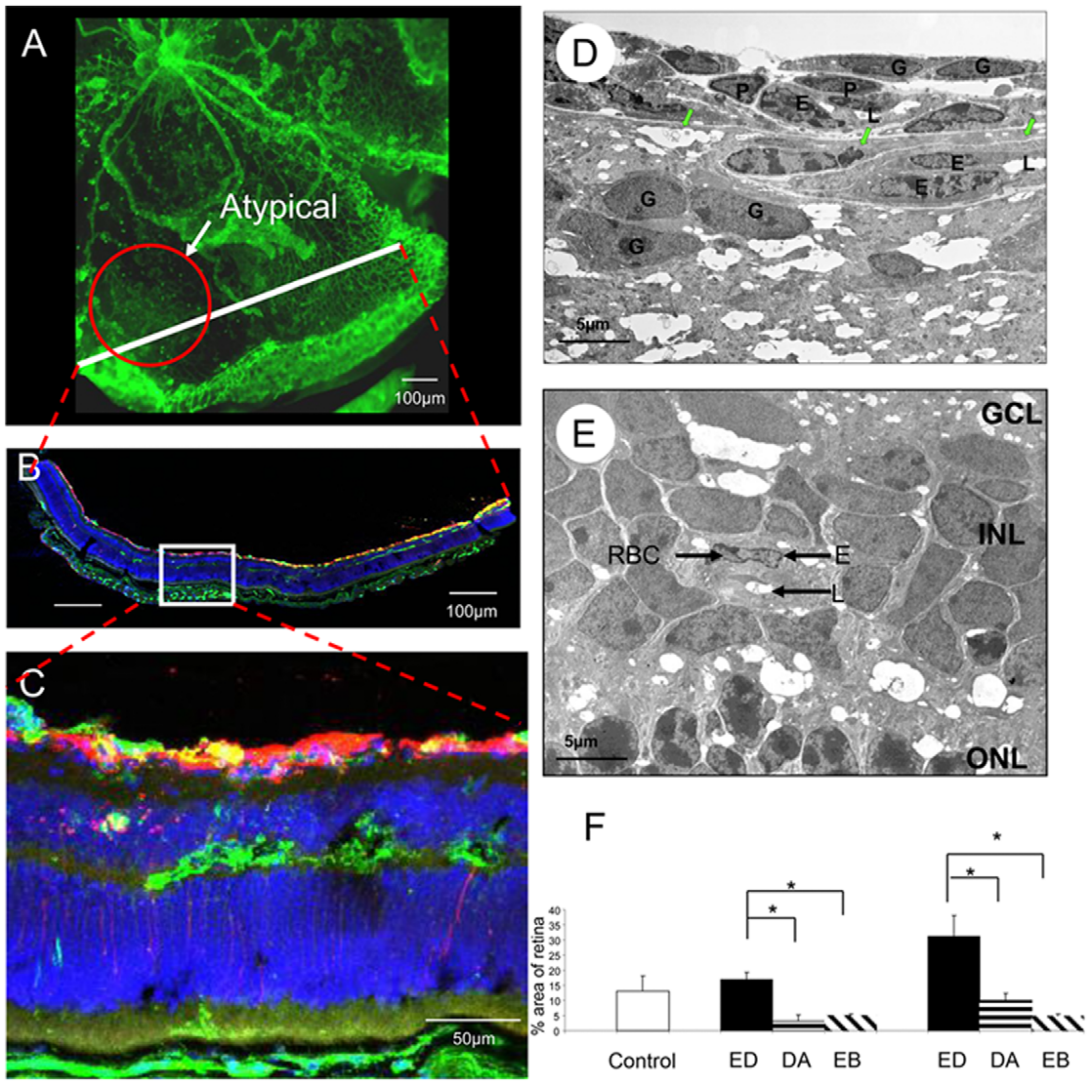
Epoetin delta modulates intra-retinal neovascularisation. The retina in Epoetin delta-treated mice often demonstrated lectin-stained “atypical vessels” (A). This is a dismounted flatmount imaged immediately prior to cryobedding. A representative of the serial sections of the flat-mounts is illustrated in Fig. (B). The area within the white box of Fig. 7B is more clearly illustrated in Fig. 7B Here it is clear that the “atypical vessels” are located inside the retina with lectin stained green and GFAP red (C). Using electron microscopy it is evident that the neovascular tissue and glial cells were on both sides of the internal limiting membrane (green arrows) (D). The pre-retinal vessels often had pericyte covering (P) and multiple glial associations (G) although both intra- and preretinal vessels had well-formed lumens (L). In the middle of the ischaemic regions there were exclusively intra-retinal vessels in the nerve fibre layer and sometimes between the Inner nuclear layer (INL) and outer nuclear layer (ONL) that were perfused with red blood cells in the lumen (RBC) (E). Epoetin delta treatment at high dose significantly increased “atypical”, intra-retinal vascularisation in OIR (*p<0.05) (F). Low dose displayed no difference in the “atypical”, intra-retinal vascularisation of the entire retina. Darbepoetin Alfa and Epoetin Beta reduced the intra-retinal vascularisation of the retina (p<0.05) (n = 7). (Error bars  =  Standard Error of the Mean).

ESA-treatment of OIR did not have a significant impact on retinal ischaemia (as observed in Figure 6A–F) as quantified at P17 (Figure 6G). During the ischaemia analysis, it was observed that the epoetin delta treatment caused a qualitative increase in lectin-positive vasculature that was completely distinct from the hyperfluorescent pre-retinal neovasculature. On flat-mount this “atypical” vasculature, was less fluorescent and the tubes were considerably more dilated and less branched when compared to the other retinal blood vessels (Figure 7A). From the flat-mount it was impossible to determine if these atypical vessels were pre-retinal in nature, but serial sections of the corresponding areas in flat-mount demonstrated that these vessels were intra-retinal, being localised to the nerve fibre layer and, especially, the outer plexiform layer (Figure 7B & 7C). Electron microscopy revealed that these atypical vessels were within the retina and had immature basement membranes and sometimes lacked covering by mural cells but they were often perfused, as evidenced by erythrocyte profiles (Figure 7D & 7E). Calculating this intra-retinal (atypical) vasculature on flatmounts with the corresponding mapping from the serial sections, it was demonstrated that epoetin beta or darbepoetin alfa treatments reduced the numbers of these intra-retinal vessels in comparison to OIR controls (Figure 7F). By contrast, epoetin delta significantly increased these vessels during OIR (p<0.05) (Figure 7F).

**Figure 8:**
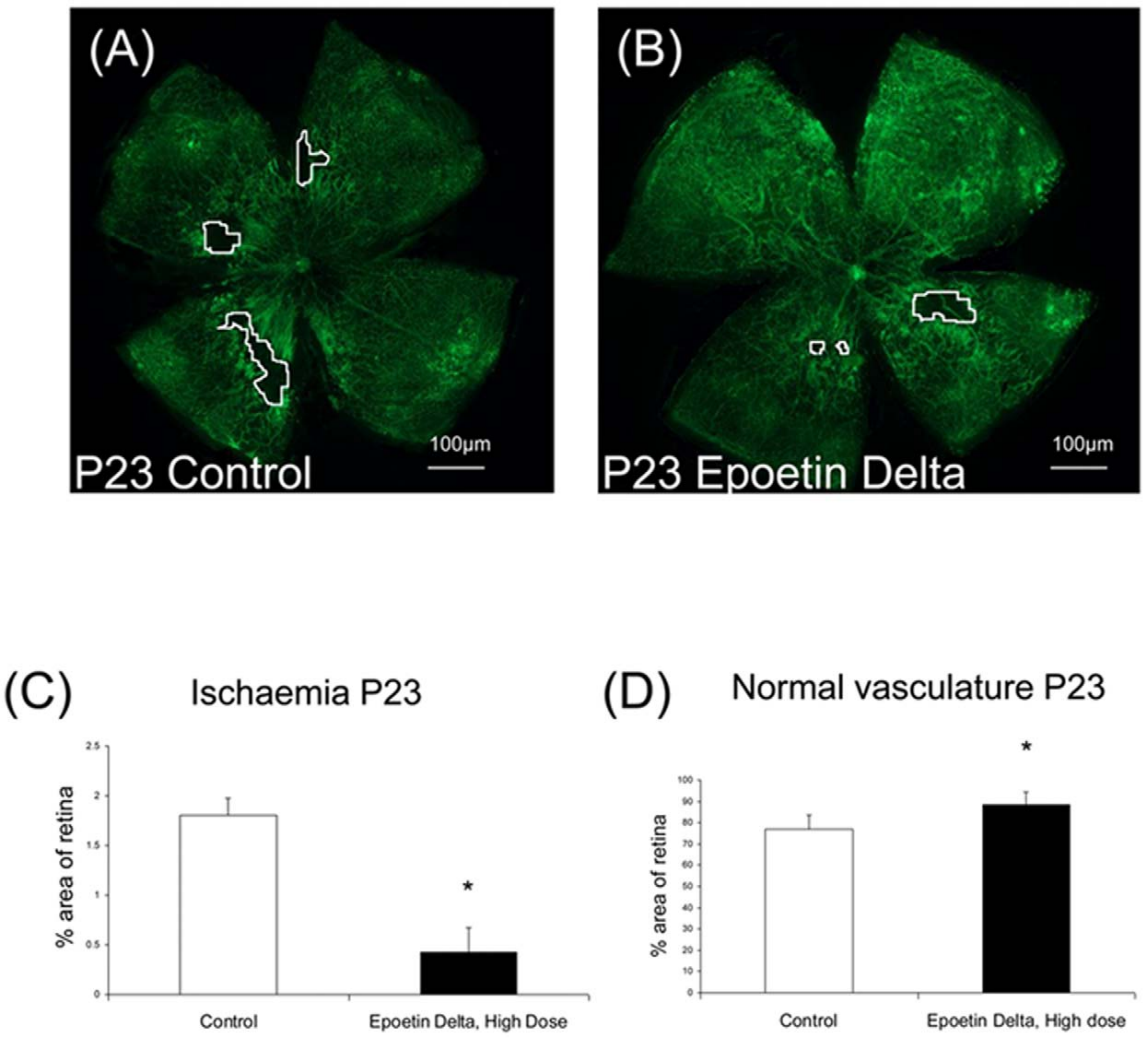
Epoetin delta enhances vascular recovery following OIR. Figs. 8 (A and B) illustrate the recovery of the vasculature in the retina and the ischaemic regions at P23 following OIR for formulation buffer treated mice and those which were treated with high dose of epoetin delta. Upon quantification the ischaemic area is decreased in epoetin delta treated mice relative to the formulation buffer treated animals (C) (*p<0.05) (n = 6). Inversely related to ischaemia, the area of normal vasculature is increased at P23 following epoetin delta treatment (D) (*p<0.05) (n = 6) (Error bars  =  Standard Error of the Mean).

In a parallel study, OIR mice were treated with epoetin delta or vehicle control (Fig. 8A & 8B) and maintained up to P23 to determine if the increase in intra-retinal vasculature observed at P17 with epoetin delta helped the retinal microvascular recovery. There was a significant reduction in the ischaemic area of the epoetin delta-treated mice compared to controls (p<0.05) (Figure 8C). Epoetin delta-treated mice also showed enhanced areas of normal vasculature at P23 (p<0.05) (Figure 8D). The ratio of normal vasculature:ischaemic retina was calculated for epoetin delta-treated and control animals. The enhanced “normalisation” of the retina was also reflected in this ratio; 210∶1 (epoetin-treated) compared to 43∶1 (formulation buffer treated). This equated to a ∼5 fold enhancement of normal vasculature at P23 following epoetin delta treatment.

### Haematopoietic stem cells in marrow and retina

**Figure 9:**
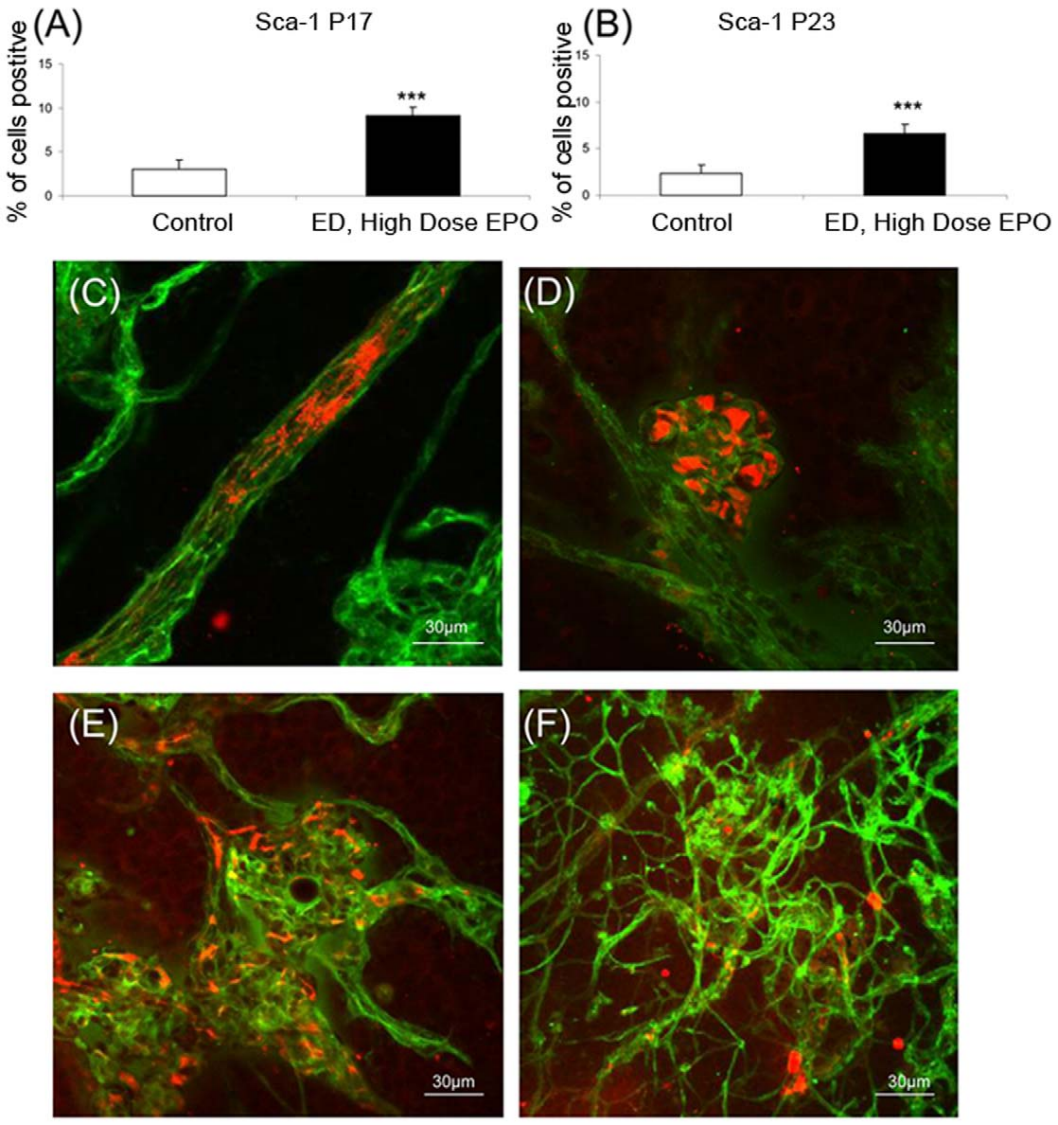
Epoetin delta stimulates haematopoetic stem cells in bone marrow and infiltration into the retinal vasculature. Sca-1 is a marker for haematopoetic stem cells (HSCs) and this was significantly enhanced in the marrow of mice treated with epoetin delta, both at P17 (A) and also P23 (B). (n = 5/group) ***p<0.001 (Error bars  =  Standard Error of the Mean). Sca-1 immunoreactivity in retinal flat-mounts demonstrated that this HSC marker was present in cells localised to intra (C) and pre-retinal blood vessels (D). Assessment of the retinal vasculature penetrating the ischaemia retina at P23 demonstrated that these vessels contained many Sca-1 positive cells (E). As with P17, these cells were also in clusters of pre-retinal neovessels at P23 (F).

To determine if epoetin delta stimulated haematopoetic stem cell (HSC) generation in bone marrow and if these cells appear in the retinal microvasculature, the proportions of Sca-1^+ve^ cells were assessed by flow cytometry in fresh marrow isolates and by immunohistology in the retina. After 5 days treatment at P17 (P12–P16 Epoetin Delta injections), the number of SCA-1 positive (Sca-1^+ve^) cells was significantly increased by epoetin delta relative to the control (9.13% vs. 3.1%; p<0.001) (Figure 9A). At P23 following continued treatment with epoetin delta, there was still a significant difference when compared to control (Figure 9B).

Sca-1 positive cells were abundant in the retina of epoetin delta-treated OIR animals (Figure 9C–F). Sca-1^+ve^ cells were observed in the blood column (Figure 9C) and they were particularly apparent in the pre-retinal neovasculature (Figure 9D) and also within and around the “intra-retinal” vessels inside the retinal neuropile (Figure 9E, F).

### Retinal microglia and cytokine expression

**Figure 10:**
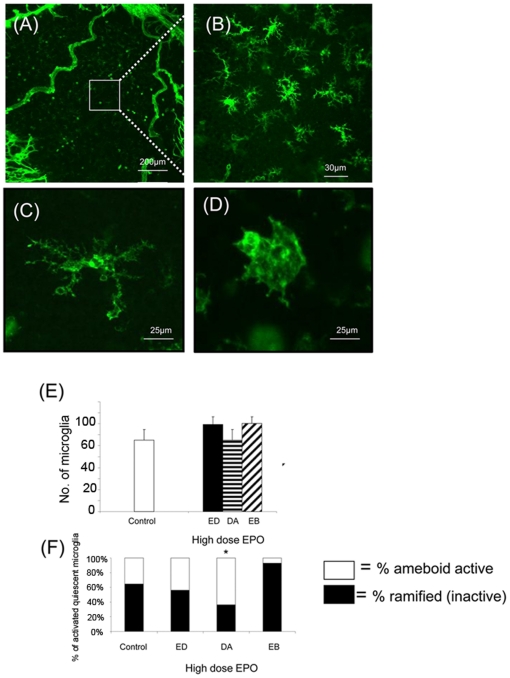
Alterations in activity of microglia in ischaemic area of the retinas of P17 ESA treated mice. Microglia were assessed in the ischaemic area of the retina (A and B) and divided into two groups – ramified (C) and ameboid (D). The activity of the microglia in the retina was assessed in the high dose of epoetin delta, darbepoetin alfa and epoetin beta. (E) There was no overall alteration in the number of microglia with Epoetin Delta (ED), Darbepoetin Alfa (DA) or Epoetin Beta (EB) administered relative to the control (n = 5 retinas assessed, four ischaemic areas per retina). In (F) The high dose of Darbepoetin Alfa increased the number of ramified microglia relative to the control (*p<.05*) (Error bars  =  Standard Error of the Mean).

**Figure 11:**
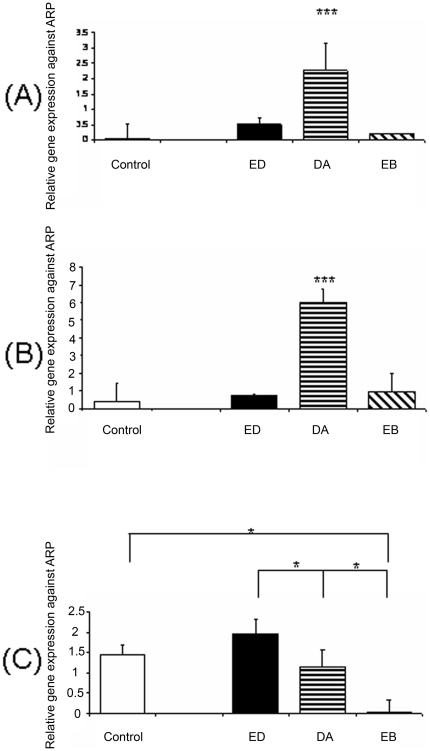
The concentration of EPO administered i.p. alters growth factors and cytokines mRNA in the retina. (A & B) TNF-alpha and VEGF was higher with darbepoetin alfa relative to the control. (n = 6***p<0.001). (C) IL-10 was reduced with EPO treatment only with Epoetin Beta. (n = 6) (*p<0.05;) (Error bars  =  Standard Error of the Mean).

Microglia were assessed in the ischaemic retina (Figure 10A &B) and were divided into two morphological groups defined as ramified (inactive) (Figure 10C) or amoeboid (active) (Figure 10D) [30]. The activation state of the microglia in the retina was assessed in epoetin delta, darbepoetin alfa and epoetin beta treated animals and in comparison to the control, there was no alteration in the total number of microglia (Figure 10E). However, darbepoetin alfa increased the number of active microglia relative to the control (p<0.05) (Figure 10F). TNF-α and VEGF mRNA was higher with darbepoetin alfa relative to the control (Figure 11A,B) (n = 6; p<0.001) and the anti-inflammatory cytokine IL-10 was reduced with Epoetin Beta. Epoetin Delta was higher than Darbepoetin Alfa and Epoetin Beta (Figure 11C) (n = 6; p<0.05).

### Summary of the effects of Epoetin Delta, Darbepoetin Alfa and Epoetin Beta

A summary of the effects of Epoetin Delta, Darbepoetin Alfa and Epoetin Beta is located in Table 2.

**Table 2:** Summary of the effects of Epoetin Delta, Darbepoetin Alfa and Epoetin Beta.

	Epoetin Delta	Darbepoetin Alfa	Epoetin Beta
**Derivation**	Human cells	Chinese Hamster Ovary cells	Chinese Hamster Ovary cells
**Glycan profiling**	Only 3 tetra – antennary peaks	4 extra Bi and Tri antennary peaks, also 2 extra tetra-antennary peaks compared to Epoetin Delta	5 extra Bi and Tri antennary peaks, also 1 extra tetra-antennary peaks compared to Epoetin Delta
**Angiogenic effect** **at 1 IU ** ***in vitro***	No	Yes	No
**Angiogenic effect** **at 20 IU ** ***in vitro***	No	Yes	No
**Angiogenic effect at 100 IU ** ***in vitro***	Yes	No	Yes
**Effect on Reticulocytes in vivo**	Low dose YesHigh dose - Yes	Low dose – NoHigh dose - Yes	Low dose - NoHigh dose - Yes
**Induce Ischaemia ** ***in vivo*** **?**	Low dose – NoHigh Dose - No	Low dose – NoHigh Dose - No	Low dose – NoHigh Dose - No
**Induce Intra-retinal Vasculature ** ***in vivo*** **?**	Low dose – NoHigh Dose - Yes	Low dose – NoHigh Dose - No	Low dose – NoHigh Dose - No
**Induce pre-retinal vasculature ** ***in vivo*** **?**	Low dose – NoHigh Dose - No	Low dose – NoHigh Dose - No	Low dose – YesHigh Dose - Yes
**Alteration in number of microglia in the ischaemic retina**	No	No	No
**Increase in the number of active microglia in the ischaemic retina**	NO	No	Yes
**Cytokine induction response ** ***in vivo*** **VEGF** **TNF-alpha** **IL-10**	NoNoDecreased	NoNoNo	YesYesNo

## Discussion

It is well established that recombinant EPO has pro-angiogenic properties *in vitro* and *in vivo* [32], [33]. In the current investigation, it has been shown for the first time that the ESA, epoetin delta, also possesses pro-angiogenic properties but that these differ appreciably from other ESAs used clinically. The observed EPO-mediated angiogenic response is different between epoetin delta, darbepoetin alfa and epoetin beta and is dose-dependent. This is reflected in the distinct differences between the glycosylation profile of each ESA studied. Unlike other ESAs studies, epoetin delta appears to have significant tissue-protection properties for ischaemic retinopathy.

Recombinant EPO induces a dose-dependent increase in endothelial cell proliferation with concentrations as low as 5 IU/ml [34]. Epoetin delta also displays a similar dose response, but it is interesting that there is a lack of angiogenic effect at <20 IU/ml and a significant induction at concentrations ≥20 IU/ml. We have similarly shown that significant in vivo responses also require higher concentrations of ESAs. It has been demonstrated that low dose EPO is sufficient for erythropoietic effects while higher doses are required for tissue protective effects and Brines and Cerami have suggested that this response may be linked to differential affinity of EPO-R and BCR for EPO [35]The high-affinity of EPO-R establishes erythropoiesis at low concentration ranges while the low-affinity of BCR typically requires higher concentrations of EPO (>500 IU/Kg) to provoke tissue protection [35]. We have demonstrated that epoetin delta in particular induced erythropoiesis at low and high doses but angiogenesis was only induced by higher doses. EPO-R is expressed by microvascular endothelial cells [34] and ligand binding is likely to evoke signalling through the MAPK and PI3K/Akt pathways [36] [37]. In these cells there is also possible involvement of the βCR ^[2]^ although this was not evaluated as part of this study.

In the *in vitro* angiogenesis system used in the present study, it was apparent that darbepoetin alfa was more pro-angiogenic at low concentrations than the two epoetins. In the in vitro angiogenesis system used in the present study, it was apparent that darbepoetin alfa was more pro-angiogenic at low concentrations than the two epoetins. Such a biphasic angiogenic effect has been previously speculated for Darbepoetin Alfa [20] similar to what occurs with VEGF. This could help explain what we have observed with Darbepoetin Alfa.

It was also apparent that VEGF neutralisation significantly reduced EPO-induced angiogenesis, indicating that this response is mediated, at least in part, by endothelial-derived VEGF. It has been demonstrated that recombinant EPO can induce VEGF expression in neural progenitor cells which, in turn, can induce an angiogenic response [37]; however, in the endothelial cell monoculture, it would appear that EPO can induce angiogenesis through VEGF autocrine stimulation, although the precise signalling mechanisms involved remain uncertain. VEGF is a recognised driver for retinal angiogenesis and such a response, if it occurs *in vivo*, would have important implications for disease-related elevations in EPO and for VEGF neutralisation therapy. This is supported by a recent report in a model of myocardial infarction in which EPO increased VEGF protein expression and improved revascularisation of the ischaemic myocardium; administration of VEGF-neutralizing antibodies prevented this EPO-mediated enhancement of cardiac revascularisation [38].

Haematocrit levels were quantified in this study but there was no alteration in the blood of the mice which were treated for 5 days (P12–16) and then sacrificed at P17. It takes 10 days to alter the haemocrit level. Therefore, in this study the percentage of reticulocytes were analysed as an early indicator for erythrogenesis and these cells were increased by both low and high dose of Epoetin delta and only the high dose of Darbepoetin alfa and Epoetin beta increased the reticulocytes. These findings suggest that the human cell derived Epoetin delta is more effective at increasing the reticulocytes in mice. This should be tested in humans also as Epoetin delta could be a more effective treatment.

An important objective for the current study was to determine the effects of three ESAs on the risk of pre-retinal neovascularisation which is a major sight-threatening end-point of many ischaemic retinopathies. We demonstrated that Epoetin delta and Darbepoetin alfa produced no significant exacerbation of pre-retinal neovascularisation, even at the highest dose. Epoetin beta on the other hand, did show an increase in pre-retinal neovascularisation compared to controls. The differences between recombinant EPOs in relation to their potential to induce aberrant retinal neovascularisation is also demonstrated by a previous study in which OIR mice were treated with epoetin alfa (at a dose of 5,000 IU/Kg) at comparable time-points to those observed in the current study and showed enhanced pathological neovascularisation [18]. The lack of pathological neovascularisation in treatment with epoetin delta is therefore unexpected, especially since it has been demonstrated that raised endogenous EPO levels are associated with proliferative diabetic retinopathy [20], [39]. Also in the OIR model, Watanabe *et al* have suggested that inhibition of EPO receptor signalling using a soluble receptor peptide delivered as an intravitreal injection prevented pre-retinal neovascularisation [20]. Therefore the differences in angiogenic potential observed between the recombinant EPOs Darbepoetin Alfa and Epoetin beta and the human cell derived Epoetin Delta in the present study may reflect a ligand-mediated modulation of the Epo receptor with differential downstream signalling outcomes and suggests that the potential risks of administering an ESA to patients with proliferative retinopathies [18], [20] may be avoided by careful choice of the EPO mimetic.

An important outcome of the current investigation is that treatment with epoetin delta significantly enhances intra-retinal neovascularisation, a response that was distinctive from other ESA treatments. The intra-retinal new vessels observed in epoetin delta-treated mice were spatially and morphologically distinct from the pathological vessels above the internal limiting membrane of the retina. It is pertinent to note that intraretinal neovascularisation is not necessarily a beneficial response and in some cases telangiectatic vessels or retinal angiomatous proliferation (RAP) pathology are linked to retinopathies. However, the intra-retinal vessels observed following epoetin delta treatment appeared morphologically dissimilar to RAP described for example in the retina of Vldlr-/- mice [40] The intra-retinal capillaries described in the current study were newly formed, reflected by their electron-lucent immature basement membranes and incomplete coverage of pericytes. They were also perfused as indicated by the presence of erythrocytes in their lumena. Extended epoetin delta treatment to P23 demonstrated an absence of these intra-retinal vessels and the retinal vasculature from treated mice appeared more normal. Therefore such vessels could to contribute to re-vascularisation of the ischaemic central retina during OIR and promote significant normalization of the vasculature when compared to controls. Intra-retinal neovascularisation can often be a highly beneficial phenomenon in OIR because it can reduce retinal hypoxia and thereby reduce the stimulus for pathological neovascularisation [29], [41].

It has been previously demonstrated that treating mice with recombinant EPO in the early stages of OIR (P6–12) prevents the vaso-obliterative stages of this pathology, possibly through mobilization of endothelial progenitor cells [18]. Janmaat *et al.* also treated mice with epoetin delta and found comparable numbers of circulating HSCs [42]. This phenomenon is also reflected in our study, in which epoetin delta treatment leads to an increase in HSCs, their mobilization in the bone marrow and the appearance of early markers for endothelial progenitors in the retinal neovasculature. EPO is a potent mobilization factor for HSCs [43], [44] and they can be harnessed to re-vascularise the ischaemic retina in OIR [40]. A recent study has indicated that EPO can improve cardiac revascularization by mobilization of EPCs and their participation in microvascular regeneration, but only when ischaemia is present [38]. A similar phenomenon could be at play in the retina and EPCs appear to be critical for the observed re-vascularisation of non-perfused retina, although this requires further investigation.

Microglia are considered the resident immune cells of the central nervous system and while they may maintain tissue homeostasis they are also involve in inflammation and neurovascular dysfunction. Retinal microglia are directly involved in cytokine expression in response to ischaemia and may regulate angiogenesis in the neuropile [40], [45]. In this study, a higher proportion of active microglia was observed with Darbepoetin alfa compared to Epoetin delta and Epoetin beta and this may be associated with the higher expression levels of VEGF and TNF-alpha mRNA in retina relative to Epoetin beta or Epoetin delta. In addition, in the current investigation, epoetin delta increased the intra-retinal vasculature although it did not alter the number of microglia within the retina nor induce appreciable upregulation of TNF-alpha or VEGF. There was a higher level of epoetin delta induced expression of the anti-inflammatory cytokine IL-10 compared to darbepoetin alfa and epoetin beta. IL-10 has been previously shown to suppress pathological retinal angiogenesis [46] and reduce retinal inflammation [47] in murine models and it is possible that epoetin delta could augment expression of this cytokine in the retina, as has been shown for other EPO-treatment of other ischaemic tissues [48].

There is no direct clinical evidence that systemic EPO can promote accelerative retinopathy in people with diabetic renal failure. However, Watanabe et al., 2005 [20]suggests that the level of EPO in the vitreous fluid of patients with proliferative diabetic retinopathy is higher than the level in diabetic patients. Indeed, EPO was more strongly associated with proliferative diabetic retinopathy than was VEGF. However, there was no significant correlation observed between the vitreous and plasma levels of EPO so increased levels in the vitreous could be due to increased local production in the retina. Li et al., (2010) [49] has tested the effects of three intravitreal injections of erythropoietin therapy for patients with chronic and progressive diabetic macular edema. They have stated the case study shows a short term positive response to EPO where their visual acuity was improved for the patients assessed with chronic diabetic macular edema. The patients were only assessed up to 18 weeks after the first injection and there was no mention of the EPO treatment promoting accelerative retinopathy but the patients were only assessed for 18 weeks. Lappin et al., 2007 [24]has stated that additional research is needed urgently to determine the effects of ESAs on risk thrombosis, rate of tumour growth and neovascularization in vivo and in vitro. The DAHANCA report 2007[50] has stated that the administration of Darbepoetin Alfa results in greater mortality than with placebo while patients who receive Epoetin Beta have poor loco-regional progression of tumours (Henke et al., (2003) [51].

In summary, this investigation has demonstrated that ESAs have distinct and significant impact on angiogenesis *in vitro* and *in vivo*. Such apparently enigmatic responses are closely linked to dosing regimen and stage of disease and even the same recombinant EPO can induce remarkably different responses when delivered prior to or at the onset of hypoxia [18]. Such differences may also reflect distinct biological properties derived from different post-translational modifications such as glycosylation which are governed by the manufacturing process of these ESAs. The disparity between the human cell derived epoetin delta and the recombinant EPOs should be carefully considered when prescribing ESAs in patients who are at risk for ischaemic retinopathy.
